# Comparing the running subcuticular technique versus the Donati technique in open carpal tunnel release: a randomized controlled trial

**DOI:** 10.1186/s13018-021-02710-0

**Published:** 2021-09-17

**Authors:** Sitthiphong Suwannaphisit, Wachirakorn Aonsong, Porames Suwanno, Varah Yuenyongviwat

**Affiliations:** Department of Orthopedics, Faculty of Medicine, Prince of Songkla University, 15 Karnjanavanich Road, Hat Yai, Songkhla, 90110 Thailand

**Keywords:** Carpal tunnel, Scar, Scar evaluation, POSAS, Wound closure, VAS score, Functional outcome

## Abstract

**Background:**

There are various skin suture techniques for wound closure following carpal tunnel release, and well-performed suturing will result in low post-operative scar tenderness. The aim of this study was to compare the Donati suture technique and running subcuticular technique in terms of surgical scar, post-operative pain and functional outcome in open carpal tunnel release.

**Methods:**

One-hundred forty-two patients were randomized using a computer-generated random number table into two groups receiving either running subcuticular suturing or Donati suturing after surgical intervention. We evaluated postoperative scarring using the Patient and Observer Scar Assessment Scale (POSAS), pain intensity using a verbal numerical rating scale, and functional outcomes using the Thai version of the Boston Carpal Tunnel Questionnaire after surgical decompression for carpal tunnel syndrome at 2, 6, and 12 weeks. Continuous data are reported as mean ± SD while normally distributed or as median (interquartile range) when the distribution was skewed.

**Results:**

Lower scores at 2 weeks were given by the patients receiving the running subcuticular suture technique than the Donati suture technique (15.3 ± 4.8 vs 17 ± 4.6, respectively, *P* < 0.05) while the observer scores were not significantly different (15.6 ± 5.8 vs 16.7 ± 5.2, respectively, *P* = 0.15). At both 6 and 12 weeks post-surgical decompression both patient and observer scores were not significantly different. There were no differences between the groups in terms of VNRS pain scores and functional Boston Carpal Tunnel Scores at all time points.

**Conclusions:**

This randomized controlled trial found that although scarring assessments were slightly better in the earliest period following wound closure after surgical decompression in carpal tunnel syndrome using the running subcuticular suture, the final results at 3 months postoperative were not significantly different.

**Trial registration:**

The study was registered at https://www.thaiclinicaltrials.org/ (TCTR20191204002).

## Introduction

Carpal tunnel syndrome (CTS) is the most common nerve entrapment syndrome [1]. The treatment is always either surgical or conservative**.** There is no other option. Patients with moderate/severe or deteriorating symptoms following conservative treatment, or with sudden and severe symptoms, are recommended to be referred for consideration of surgery [2].

Surgery-related complications include tender scar, persistent symptoms, neurovascular injury, wound complications, bleeding, pillar pain (a deep aching pain at the base of the thenar eminence and across the wrist), and/or reduced grip strength. Most of these occur only rarely, in the order of <1%; however, scar tenderness and pillar pain are reported in 7% and 18% of patients, respectively, sometimes; persistent pain [3]. Post-operative scar tenderness might be caused from an inversion of the wound edges.

There are various skin suture techniques for wound closure following carpal tunnel release, and well-performed suturing will result in low post-operative scar tenderness. To date, no studies have attempted to define the ideal suture technique for carpal wound closure. The Donati suture technique aids in precise skin edge eversion [4], while the running subcuticular sutures technique has the advantage of closing wounds with equal tissue thickness [5], and virtually no tension exists, which leads to better cosmetic results [6]. However, to date, there are no studies comparing these two techniques in terms of aesthetic outcome, post-operative pain, and functional score. Therefore, the primary aim of this study was to compare the aesthetic outcomes between these techniques. Secondary outcomes measured included post-operative pain and functional outcomes.

## Patients and methods

### Study design

This study was a prospective, randomized, controlled, interventional (Donati suture and running subcutaneous suture), single-blinded trial (the outcome assessors were blinded) performed at one tertiary center in Songkla, Thailand. The local institutional review board approved the study protocol (IRB number EC 61-405-11-1), and the procedures in this study were performed under the Declaration of Helsinki’s ethical principles for medical research involving human participants. Written informed consent was obtained from all individual participants included in the study. The study was registered at Clinicaltrials.in.th (TCTR20191204002).

### Recruitment

We enrolled adult patients with carpal tunnel syndrome, aged between 18 and 99 years, based on a combination of the patient’s history (pain, paresthesia, and/or hypotheses in the hand in the area innervated by the median nerve), physical examination (thumb abduction weakness: thenar atrophy), and measurements of nerve conduction velocity.

The inclusion criteria for surgery consisted of failed conservative treatment after treatment of 6 months (patients still having persistent clinical pain and/or paresthesia), who presented with thenar atrophy or thumb abduction weakness at their first visit, or with severe compression following an electromyography (EMG). Between March 2018 and December 2020, all patients aged > 18 years with carpal tunnel syndrome were recruited. The exclusion criteria were (1) history of lidocaine allergy, (2) history of wrist trauma or previous wrist operation, (3) previous diagnosis of cervical spondylotic radiculopathy, (4) patients who developed a drug allergy during the study period and needed to be admitted, and 5 on clopidrogel or warfarin.

### Randomization and blinding

The patients were randomly allocated to either the Donati suture technique (group I) or running subcuticular suture (group II) group. Block-of-four randomization with computer generated random numbers was used for allocating the patients into the two groups. The envelopes were opened in the operating room just before wound closure.

All other hand-surgeon hospital staff responsible for treating the participants after surgery were blinded to the allocation. The investigator opened a sealed opaque envelope containing the allocation code after completing surgical decompression, when preparing to suture the surgical wound. Closed the wound using the method inside the envelope.

The participants were blinded to the suturing method throughout the study period. They underwent the same aftercare and follow-up regimens regardless of the treatment group, and the investigator who administered the treatment did not participate in the follow-up examinations.

### Interventions

**Fig. 1:**
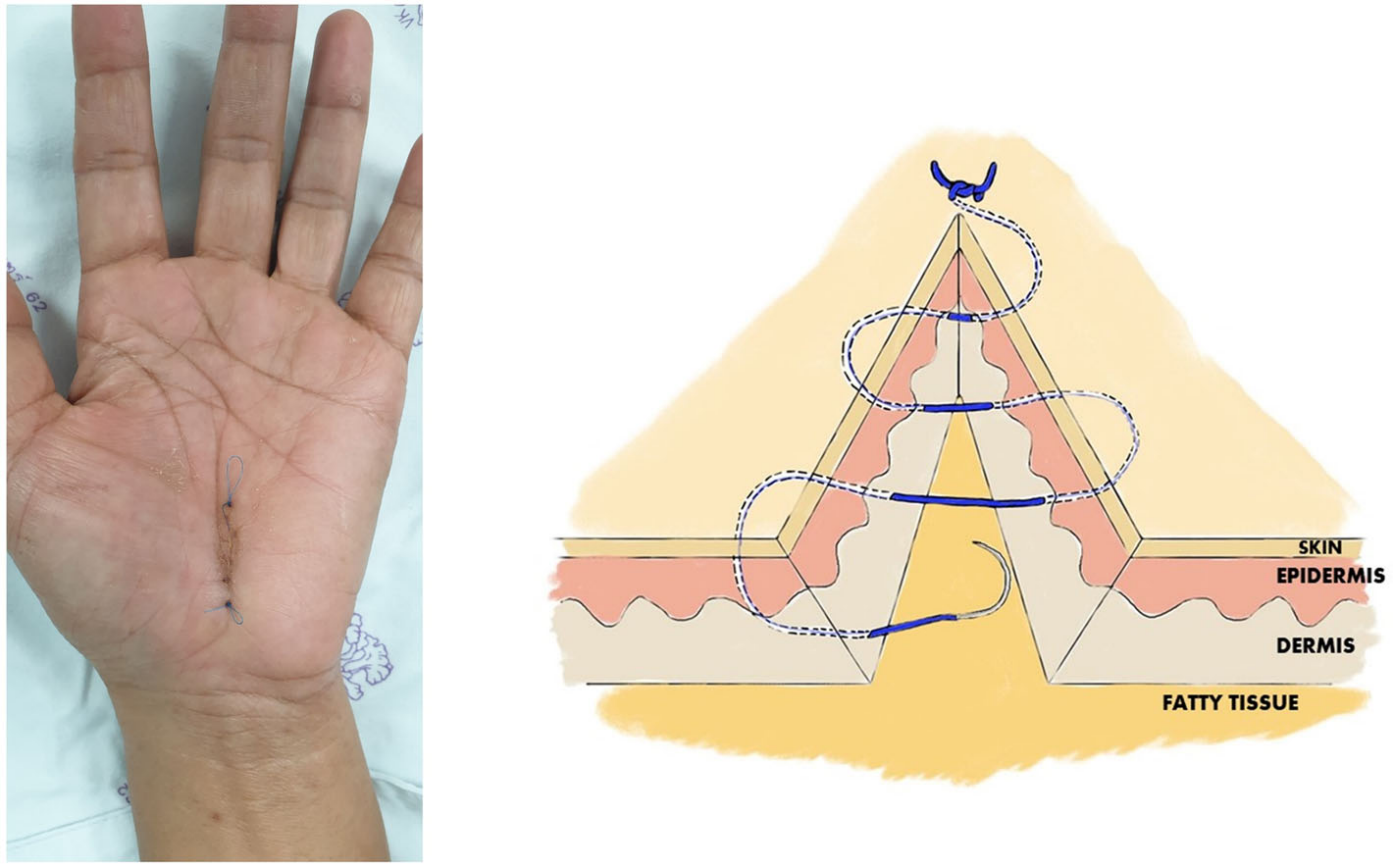
A clinical photograph and drawing picture showing wound closure using the running subcuticular technique

**Fig. 2:**
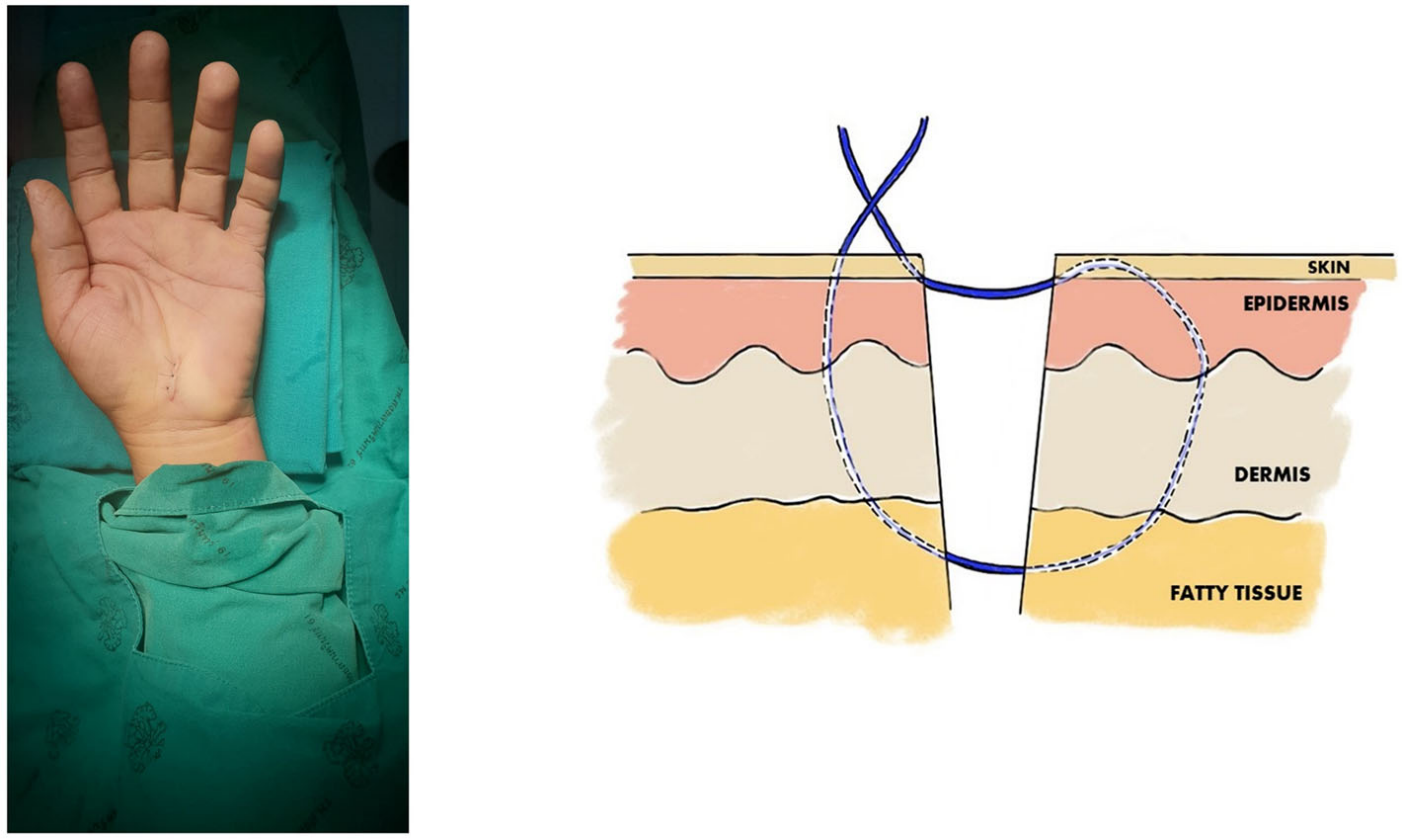
A clinical photograph and drawing picture showing wound closure using the Donati technique technique

All procedures were performed under local anesthesia and under tourniquet control, using lidocaine 2% with adrenaline 1: 100,000 into the palmar soft tissues and the carpal tunnel itself. For each procedure, a skin incision of approximately 1.5 cm was made, in line with the radial half of the ring finger ray, and not crossing the wrist flexor crease. A No. 15 scalpel was used for opening the skin and transecting the carpal ligament in the proximal direction, and Metzenbaum scissors were used to cut the carpal ligament in the distal direction. No epineurotomy or any other form of neurolysis was performed. The skin was sutured using nylon 4-0, with two different techniques depending on the randomization group of each patient, and a compressive bandage was applied for 2 days. In the running subcuticular technique group, skin closure was begun by inserting a needle through one wound edge. The opposite edge was everted and the needle was placed horizontally through the upper dermis; this was repeated on alternating sides of the wound. The suture was terminated similarly to the running subcutaneous suture at the distal end of the wound (Fig. 1). For the Donati technique group, skin closure started near the wound edge (1–3 mm), by inserting the needle into the deeper aspect of the opposing side, and exiting through the epidermis wide to the insertion (0.5–1.0 cm). Then, the needle was reversed and re-inserted into the skin 1–3 mm from wound edge (1–3 mm) of the side just exited, and the remainder of the first pass repeated and exiting wide to the initial penetration (0.5–1.0 cm) (Fig. 2). Following the operation, a wrist splint was not used, and the patients were encouraged to move his or her fingers as soon as possible. All patients received naproxen 250 mg, one tablet two times daily and dicloxacillin 500 mg, one capsule four times per day for the first 5 days.

At 2 weeks postoperatively, the patient had their sutures removed by a nurse who was not involved in the study. After the sutures were removed, the patient was sent to an independent observer for scar assessment.

### Outcomes

One independent observer, who was blinded to the suturing techniques, performed assessments of each subject at 2, 6, and 12 weeks postoperatively. A scar assessment was carried out in the outpatient department at the same times using a modified Patient and Observer Scar Assessment Scale (POSAS). This scale, which has been validated for linear scars, assesses the scar from the viewpoint of both the patient and the clinician [7]. The scores range from 1 to 10 in each item. The lowest score is “1” reflecting normal skin with the highest score of 10 meaning the largest difference from the normal skin (i.e., the worst imaginable scar). And during the same visits each patient completed the Thai version of the Boston carpal tunnel questionaire [8], with lower scores reflecting a nearly normal hand and higher scores worsening symptoms. The severity of pain in the hand was measured preoperatively and 1 day postoperatively, and then at the same 2, 6, and 12-week intervals postoperatively by a verbal numerical rating scale from 0 (no pain) to 10 (worst possible pain). Sensation in the index finger and little fingers was tested before the operation and at the same time points as indicated earlier using a 2-point discrimination test.

### Study flow

**Fig. 3:**
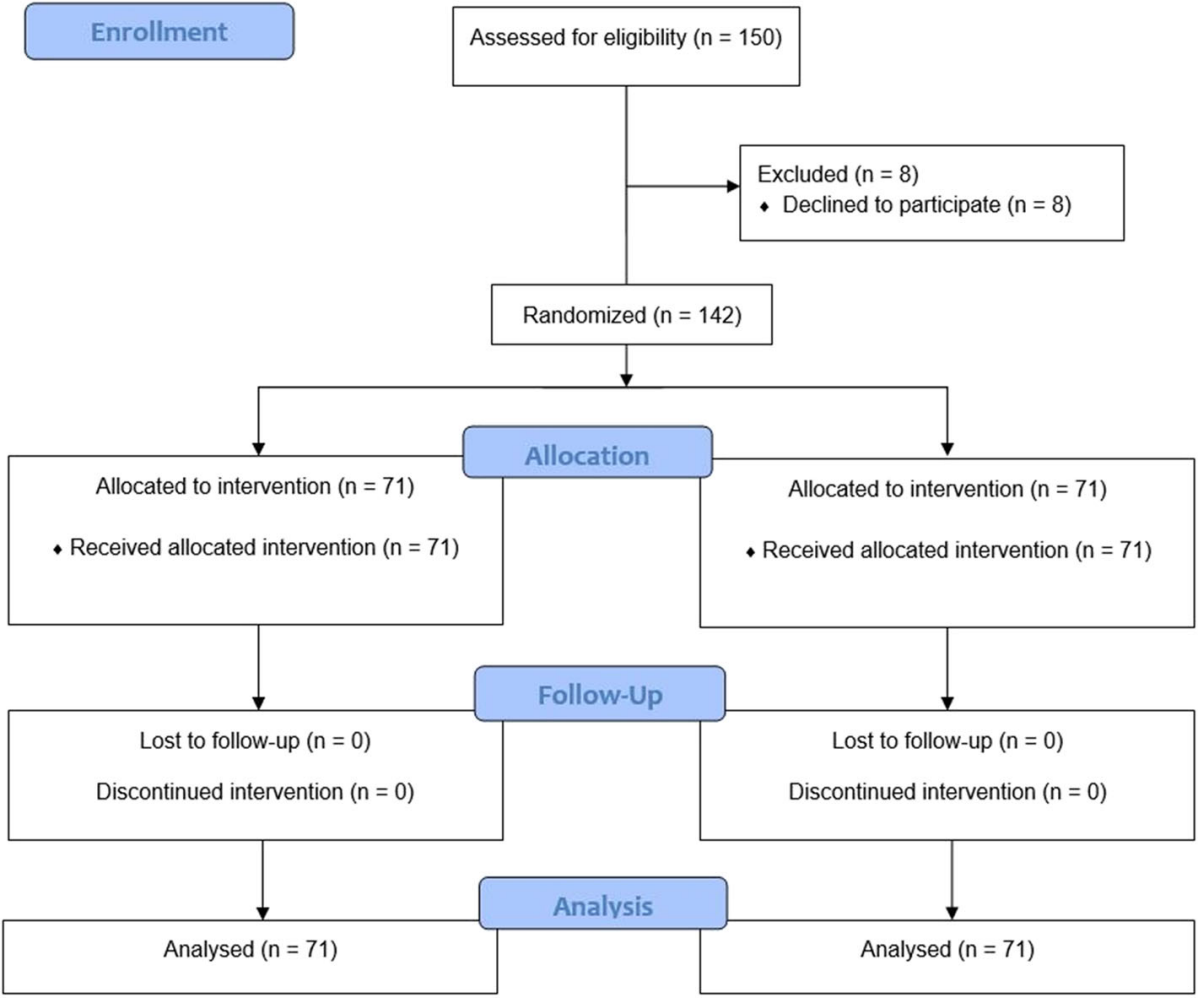
CONSORT flow diagram for the running subcuticular versus Donati suture

We screened 150 patients, eight of whom declined to participate (Fig. 3). The groups were not different at baseline with regard to age, duration of symptoms, pre-operative VAS scores for pain, DASH scores, and grip strength/pinch strength. During the follow-up period, no participants withdraw from the study or were lost to follow-up (Fig. 3). Baseline data were not different between participants who completed the study. There were no adverse effects in any patient in either group.

### Statistical analysis

For a superiority trial, with an effect size of 80% and a margin of 10%, 59 hands per group were needed (alpha 5%, power 80%). We estimated patients lost to follow up at 20%, and therefore, 71 patients were recruited per group. All participants were included in the analysis as randomized regardless of discontinuation of treatment, lost to follow-up, or treatment conversion (intention-to-treat principle). To estimate between-group differences, we calculated improvement in the VAS score for pain, POSAS score, Thai Boston Carpal Tunnel score, and grip strength from baseline to 2, 6, and 12 weeks after surgical decompression. These improvements were compared using paired *t* test. We reported group differences in improvement from baseline scores using a generalized linear mixed effect model at every timepoint. Continuous data were reported as mean ± SD when normally distributed or as median (interquartile range) when the distribution was skewed. Outcome measures were analyzed with *t* test and generalized linear mixed models. We considered a *p* value of 0.05 to indicate statistical significance. R Program Version 3.4.5 (R Foundation for Statistical Computing, Austria) was used for all statistical analyses.

## Results

A total of 142 participants affected by idiopathic CTS, 71 assigned to the Donati suture group and 71 to the running subcuticular suture group, were included in the study and underwent open carpal tunnel release from March 2018 to December 2020. Twenty-two were males and 120 were females, with age ranging from 31 to 81 years (mean 59 years). The operation was performed on the right hand in 69 patients and the left hand in 73 patients. There were no wound hematomas, infections, or dehiscence in either group, and no patients were lost to follow-up during the study. There were no significant differences between the group in age of the patients (*p* > 0.05) (Table 1) or operative characteristics (*p* > 0.05) (Table 2).

**Table 1:** Comparing patient baseline characteristics between the running subcuticular skin closure and Donati skin closure groups

Characteristic	Running subcuticular skin closure group (*n*=71)	Donati skin closure group (*n* = 71)	*P* value
Age (years)	58.1 (9.5)	60.2 (9.4)	0.192
Sex			1.34
Male	8 (11.3)	14 (19.7)	
Female	63 (88.7)	57 (80.3)	
Occupation			
Housemaid	20 (28.2)	13 (18.3)	
Government Officer	2 (2.8)	2 (2.8)	
Teacher	21 (29.6)	32 (45.1)	
Agriculturist	28 (39.4)	24 (33.8)	
Underlying disease			1.02
Yes	30 (42.3)	37 (52.1)	
No	41 (57.5)	34 (47.9)	
Current CTS problem (month)	16.6 (±20.7)	14.9 (±17)	0.54
Smoking			
Yes	3 (4.2)	3 (4.2)	
No	68 (95.8)	68 (95.8)	
Preoperative VNRS score	5 (±2.1)	4.8 (±2.1)	0.48

**Table 2:** Comparing operative characteristics between the running subcuticular skin closure and Donati skin closure groups

Characteristic	Running subcuticular skin closure group (*n*=71)	Donati skin closure group (*n* = 71)	*P* value
	Mean (±SD)	Mean (±SD)	
Mean wound length (mm)	18.1 (±2)	18.8 (±1.8)	2.06
Suturing time	4.1 (± 1.3)	3.8 (±1.2)	1.31
Tourniquet time	13.2 (±2.9)	12.6 (± 2.8)	1.19
Operative time	9.1 (2.5)	8.8 (2.5)	0.54

### Surgical scar

**Fig. 4:**
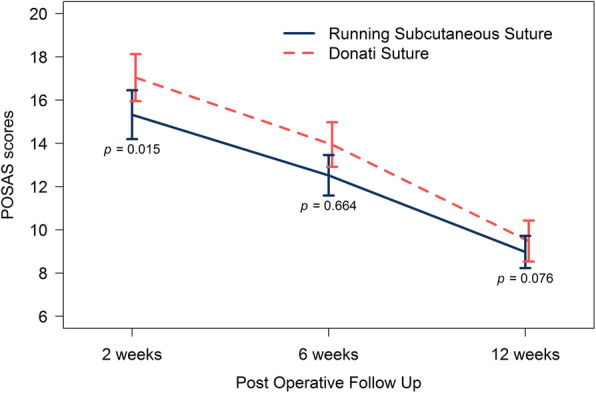
POSAS score evaluated by patient at each time point after carpal tunnel decompression

**Fig. 5:**
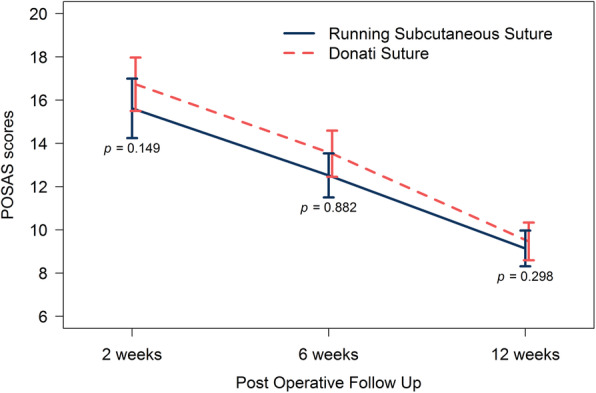
POSAS score evaluated by physician at each time point after carpal tunnel decompression

Scar assessment at 2 weeks after post-surgical decompression revealed a significant difference between these two groups in terms of patient scores (*p*<0.05). The average score was lower for the running subcuticular suture than for the Donati suture (15.3 ± 4.8 vs 17 ± 4.6; *p* < 0.05), while observer scores were not significantly different (15.6 ± 5.8 vs 16.7 ± 5.2; *P* = 0.15). The 6 and 12 weeks post-surgical decompression scores for both patients and observers were not significantly different (Figs. 4 and 5).

### Pain

We found no differences between the Donati suture and the running subcuticular suture groups in terms of improvement of VNRS pain scores at 2, 6, and 12 weeks post-surgical decompression (Table 3). The VNRS pain scores decreased in both treatment groups through the study period.

**Table 3:** Comparing operative pain score using VNRS between the running subcuticular skin closure and Donati skin closure following at 2 weeks, 6 weeks, and 12 weeks post-surgical decompression

Post-surgical decompression time	Running subcuticular skin closure group (*n*=71)	Donati skin closure group (*n* = 71)	*P* value
	Mean (±SD)	Mean (±SD)	
Baseline	5 (±2.1)	4.8 (±2.1)	0.63
2 weeks	3.1 (±1)	3 (±1)	0.56
6 weeks	0.5 (± 0.7)	0.5 (±0.9)	0.92
3 months	0 (±0.1)	0.1 (±0.3)	0.32

### Function

We found no between-group differences in functional endpoints between either the Donati suture or subcuticular suture groups at any timepoint. The Thai Boston Carpal Tunnel scores decreased in both groups during the follow-up period, and the grip/pinch strength increased in the final follow-up at 3 months after post-surgical decompression (Table 4).

**Table 4:** Comparing operative functional score using Thai-Boston Carpal tunnel score and grip/pinch strength between the running subcuticular skin closure and Donati skin closure following post-surgical decompression at 2 weeks, 6 weeks, and 12 weeks

Post-surgical decompression time	Running subcuticular skin closure group (*n*=71)	Donati skin closure group (*n* = 71)	*P* value
	Mean (±SD)	Mean (±SD)	
Boston carpal tunnel score at			
baseline	55.5 (±12.4)	57.5 (±12.4)	0.34
2 weeks	38.8 (±9.8)	38 (±7.7)	0.60
6 weeks	30.5 (± 6.8)	30.6 (±6.2)	0.97
3 months	22.8 (±4.4)	24 (±5.2)	0.14
Grip strength (pound)			
Baseline	43.6 (±19.3)	41.1 (±19.2)	0.45
2 weeks	38.5 (±17.8)	37.2 (±17.3)	0.34
6 weeks	42.3 (±16.6)	41.6 (±16.5)	0.64
3 months	50.7 (±16.6)	47.4 (±16.5)	0.24
Pinch strength (pound)			
Baseline	8.8 (±3.7)	8.7 (±4)	0.70
2 weeks	8.2 (± 3.3)	7.6 (±3.7)	0.34
6 weeks	9.3 (±3.8)	9.1 (±4.1)	0.82
3 months	11.4 (±3.8)	11.1 (±4.5)	0.69

## Discussion

Post-operative scar formation following open carpal tunnel release is one of the causes of pain. Releasing the transverse carpal ligament cause deep cutaneous injuries, which consequently lead to serious problems such as hypertrophic scars and keloids [9]. It can cause pain that can affect the patient’s quality of life, physical status, and psychological health [10]. We believe, and believed, that if the patients have good wound healing, the post-operative outcomes will be successful, in terms of cosmetic and functional results. One of the most important factors is the suturing method used to prevent hypertrophic scars and keloids as more tension around the wound tends to produce bad scars [11].

Although basic scientific knowledge concerning skin response to physical tension remains uncertain [12], mechanotransduction is the most believed theory, which states that mechanical forces are converted into biochemical responses [13]. A study by Gurtner et al. [14] on the fibrotic effects of mechanical tension supported this theory. The present study found that both the Donati suture and subcuticular running sutures resulted in low POSAS scores, and excellent scar formation. Although the Donati suture resulted in higher POSAS scores 2 weeks postoperatively, the 6-week and final follow-up scores at 3 months after the operation were not different from the subcuticular running suture scores. One study reported that the running subcuticular sutures provided equal tissue thickness and virtually no tension, which seemed superior to the Donati suture in terms of cosmetic results [6].

The secondary outcome of this study was to assess functional scores and pain scores comparing between the two closure techniques, scores which are representative of the clinical outcome of successful wound healing. We used the Boston carpal tunnel questionnaire, which is the most widely used self-administered outcome scale for the specific disease of carpal tunnel syndrome [15–17] and has also been translated into a Thai-version [8]. Pain scores were assessed using a verbal numerical scale, which has good sensitivity and is easily comprehensible for most patients [18]. Both groups reported a decrease in both the Boston carpal tunnel scores and pain scores, meaning improvement in the overall functional score. Additionally, there were no complications in either group. Based on our results, we suggest that either suture techniques is suitable for closing the wound after open carpal tunnel release.

Some studies believe that have examined the type of suture materials and their impact on the results after open carpal tunnel release [19–24]. We believe that the closure skin methods can also have an impact on the results of treatment after open surgical decompression in carpal tunnel syndrome. One previous study compared the single interrupted sutures and Donati suture methods and found that the Donati suture group had overall worse functional scores due to higher pain scores when compared with the single interrupted suture group. However, the results of the functional scores between the treatment groups did not reach statistical significance, possibly because of the relatively small sample size.

Our study is the first and the largest in the literature comparing running subcuticular suture and Donati suture in the closure of open carpal tunnel release surgery, in terms of wound assessment, functional outcome, and post-surgical decompression pain. However, the study had some limitations. First, the follow-up period was too short to assess long-term complications. For example, a 6-month follow-up would have been better to assess scar formation after carpal tunnel release. Second, we found that many patients complained of greater pain during removal of the running subcuticular suture than when removing the Donati suture. However, we did not apply a pain score during either type of suture removal, so we cannot provide any statistical analysis of this complaint. Further studies should include longer follow-ups and measure pain scores at suture removal.

## Conclusion

This randomized controlled trial found that wound closure after open CTS release using running subcuticular suture had an advantage in terms of better cosmetic results than the Donati suture at 2 weeks postoperatively. However, the cosmetic results leveled off at 6 weeks and 3 months postoperatively with no significant differences.

## Data Availability

The datasets analyzed during the current study are available from the corresponding author on reasonable request.

## Abbreviations

POSAS: Patient and Observer Scar Assessment Scale
VNRS: Verbal numeral rating scale
EMG: Electromyography
